# Mapping the Growth of Individual Placement and Support Services in Norway

**DOI:** 10.3389/ijph.2025.1608739

**Published:** 2025-09-29

**Authors:** Nils Abel Prestegård Aars, Beate Brinchmann, Miles Rinaldi, Laurent Olivier Trichet, Unni Kolstad, Kristine Sofie Steen, Cathrine Fredriksen Moe, Thomas Lorentzen, Marit Borg, David McDaid, A.-La Park, Eóin Killackey, Arnstein Mykletun

**Affiliations:** ^1^ Centre for Work and Mental Health, Nordland Hospital Trust, Bodø, Norway; ^2^ South West London and St George’s Mental Health NHS Trust, London, United Kingdom; ^3^ Centre for Research and Education in Forensic Psychiatry, Haukeland University Hospital, Bergen, Norway; ^4^ Faculty of Nursing and Health Sciences, Nord University, Bodø, Norway; ^5^ Department of Sociology, University of Bergen, Bergen, Norway; ^6^ Faculty of Health and Social Sciences, University of South-Eastern Norway, Drammen, Norway; ^7^ Care Policy and Evaluation Centre, Department of Health Policy, London School of Economics and Political Science, London, United Kingdom; ^8^ Orygen, Parkville, VIC, Australia; ^9^ The University of Melbourne Centre for Youth Mental Health, Parkville, VIC, Australia; ^10^ Department of Community Medicine, UiT - The Arctic University of Norway, Tromsø, Norway; ^11^ Division of Health Services, Norwegian Institute of Public Health, Oslo, Norway; ^12^ Centre for Population Health, Helse Bergen, Bergen, Norway

**Keywords:** individual placement and support, implementation, geographical variation, mental health, health service accessibility

## Abstract

**Objectives:**

Individual placement and support (IPS) is an evidence-based form of vocational rehabilitation that aims to help people with mental illness obtain and remain in competitive employment. The objective of this study is to quantify the national growth of IPS over an 8-year period in Norway.

**Methods:**

Using a combination of qualitative and registry data, we map how IPS was implemented in Norway between 2012 and 2019, both in terms of geographic availability and intensity of service provision.

**Results:**

In 2012 IPS was available in 4 out of 19 counties, with 14.9% of the population living in an area where IPS was present. By 2019 this had increased to all 19 counties in Norway, and more than 70% of the population lived in an area of Norway where IPS was available. The results are presented in eight heat maps that visually display how the intensity and availability of the service have expanded.

**Conclusion:**

This study has identified when and where IPS became available in Norway, which is key to future effectiveness studies of IPS in the IPSRON project.

## Introduction

As Aristotle said, “virtues are formed in man by his doing actions” [1]. As adults in any human society work is an essential part of our lives. Work is not only a means to provide for oneself and one’s relatives; it is also important for social inclusion, empowerment, self-esteem and recovery from illness [2, 3]. The right to work is a fundamental human right for those who wish to do so [4, 5]; however, individuals with mental health conditions are disproportionately affected by labor market exclusion, as these conditions are associated with disability and welfare dependency [6]. For individuals diagnosed with severe mental illness (SMI), gaining and retaining competitive employment is a challenge and is often a key goal [7–9]. Individual Placement and Support (IPS) is a supported employment model developed for individuals with mental illness. This model seeks to support individuals in gaining competitive employment while simultaneously providing the necessary mental health, social, and employment support to help them remain in employment. In essence, IPS is a “place-and-train” model, rather than the “train-and-place” approach that has dominated vocational rehabilitation for many years [10]. For individuals with mental illness, IPS has proven to be an effective approach to obtain and remain in competitive employment [11, 12] and the model is now available in more than 20 countries [13]. It is based on eight key principles, and IPS vocational units are subject to fidelity reviews to ensure adherence to these [14].

IPS first became available in Norway around 2010 and was gradually implemented across the country [15]. Funding transitioned gradually from project- and pilot-based allocations to more stable, institutional funding provided by the Norwegian Labour and Welfare Administration (Nav) [16]. According to Moe et al. [16], it took clinicians 15 years from first becoming aware of the concept to implementing it at scale. IPS is now part of the Norwegian national guidelines for the treatment of individuals diagnosed with psychosis who seek employment [17]. IPS is thus a policy, reflected in national strategies, plans and funding [18, 19]. With respect to the target group for the model, the recently published Norwegian guidelines state that IPS should be prioritized for individuals with SMI and/or substance abuse [20]. In addition, the guidelines also state that IPS should be made available for subjects with moderate mental illness requiring long-term and comprehensive work rehabilitation. In a Norwegian study on IPS conducted in 2014 [21], approximately 45% of patients had SMI and 55% had moderate mental illness. Key actors in the provision of Norwegian IPS services are the client’s clinician from mental health services, a welfare sector counsellor from Nav, Employment Specialists (ESs) and the IPS supervisors. The ES works closely with the IPS clients, supporting them in obtaining and maintaining competitive employment. To this end, the ES is expected to collaborate with the client’s potential employers, the client’s clinicians and public employment agencies [16], and thus play a coordinating role in the vocational rehabilitation process of their clients. IPS supervisors, on the other hand, ensure that IPS vocational units adhere to the eight principles of IPS, which are key to the successful delivery and efficacy of the service, and also perform fidelity reviews [22].

To achieve the policy ambition of helping more individuals with mental health conditions gain competitive employment, it is integral to public service resource planning to quantify the availability of the service that will achieve this. As an example, public services such as healthcare and education are often planned on a *per capita* basis (e.g., the number of general practitioners, teachers, etc. per 100,000 individuals), and per geographic unit (e.g., municipality level). IPS guidance from England suggests that an ES will work with 40–50 clients over the course of 1 year [23]. Accordingly, the number of clients who may receive IPS in a region depends in part on the number of ESs in that region. Developing an IPS workforce is a challenge [16, 24, 25], and an overview of how the ES workforce develops throughout the stages of IPS implementation is lacking. There is an abundance of trials demonstrating the superior efficacy [26, 27] and cost-effectiveness [28] of IPS compared to traditional vocational rehabilitation. Despite these merits little is known about whether the efficacy of IPS in controlled settings translates into societal effectiveness once it has been fully implemented. A difference-in-difference study examining the effect of IPS implementation on employment outcomes in a municipality showed promising results regarding societal effectiveness; however, the study was based on implementation in one municipality only and needs to be replicated on a larger scale [29].

Our study is part of the IPSRON project, which is led by the Centre for Work and Mental Health (CWMH) at Nordland Hospital in Bodø. The IPSRON project will investigate the societal and economic consequences of IPS implementation in Norway. Specifically, the project aims to investigate whether the implementation of IPS has had effects on outcomes such as employment, sickness absence (SA), disability benefits (DB), work assessment allowance (WAA), healthcare utilization, housing, loneliness, and crime among individuals with mental illness aged between 18 and 45. This age group was selected as it corresponded with the age of the majority of IPS clients [30]. In the present study, we examine the availability of IPS in Norwegian municipalities between 2012 and 2019. Our study is a unique opportunity to capture and develop knowledge about how the large-scale expansion of a new service develops. At the same time, we provide a means to illustrate how the service has evolved from being a limited resource to near-population coverage in Norway.

## Methods

### Setting

Our study is set in Norway, a country with a population of approximately 5.5 million inhabitants. The country is administratively organized into 15 counties, which are further divided into 357 municipalities. In 2015, the Norwegian parliament unanimously agreed on a reform of the municipalities and counties, with several merging to form new and larger units. Nevertheless, in 2024 the number of inhabitants in municipalities ranged from 215 in the smallest to 717,710 in the largest [31].

Norway is a wealthy country, with a more equal distribution of wealth than many other OECD countries [32]. Healthcare is public, free and financed through taxation. The healthcare service is organized as a two-tier, publicly funded system free at the point of access, with specialist mental health services prioritizing individuals with the most severe conditions. The specialist healthcare sector is organized into five regional health authorities. IPS ESs, however, is presently organized through local Nav offices, although this was not necessarily the case during our study period. The IPS vocational unit (ESs) is supervised by IPS supervisors who are also employed in Nav. When IPS was first implemented in Norway, it was primarily driven by clinicians working in the specialist healthcare sector who were looking for new and promising treatments for individuals with mental illness [16]. This engagement led to considerable efforts being made to gain knowledge about the IPS model, secure funds, and incorporate employment support into treatment teams. Crucially, this approach aligned with the policy ambition to prioritize functional recovery in the rehabilitation of individuals with mental illness, particularly by fostering cross-sectoral collaboration and integrating services, making IPS especially well-suited to achieving this goal [16, 33]. The national implementation of IPS in Norway has been supported by a structured system of coordination and capacity-building. The Norwegian Labour and Welfare Directorate has facilitated a national project group, regular online forums, and training initiatives, while regional IPS coordinators in each county work to sustain program fidelity locally [34].

### Design and Data Collection

This study quantified the presence and intensity of IPS service provision in Norway. To this end we first obtained from the Norwegian Directorate of Health a list of all services that had received funding for positions as ESs in the period 2012–2018. This was supplemented with a list of every service that had conducted fidelity reviews, which we obtained from a senior IPS trainer at the county level. The project was introduced in a digital briefing for all the county-level IPS trainers. Then, the trainers were sent a list of all the IPS services that we were aware of in their respective counties. The trainers were asked to quality check the list and expand it with any other known IPS services in the county, whether currently active or not. We also asked the trainers to provide a contact person for each service. Our final list included 73 IPS vocational units. The IPS supervisors were contacted and asked to participate in a semi-structured qualitative interview about the implementation of IPS. The interview guide was developed by the IPSRON research team, and is attached in Supplementary File S1. The interviews were conducted by two staff members from CWMH between 2021 and 2023.

### Data Analyses

Following the interviews, we extracted and quantified key variables for each vocational unit by year of operation. Variables derived from the interviews included the number of ESs, their locations, and the municipalities served by the health services where the ESs were integrated. We retrieved a list of all municipalities, their unique municipality numbers, and the annual populations per municipality for the years 2012–2019 from Statistics Norway [31]. We then summarized the total population served by each IPS vocational unit (for each year in our study period) and calculated the intensity of IPS, operationalized as the number of ESs per 100,000 individuals per year. After a national reform of municipalities between 2013 and 2021, the number of municipalities in Norway was reduced from 428 to 356 [35]. Since point of contact described IPS provision per vocational unit based on the municipal structure at the time of the interview, this influenced how municipal coverage was determined for each year. Specifically, if municipalities “x” and “z” were merged into municipality “y” in 2018, we considered IPS to have been available in both municipalities “x” and “z” in 2017 if our informant reported on municipality “y” at the time of the interview.

We were unable to obtain data on the number of ESs from four IPS vocational units for some of the years, either because the informant in question did not possess this data or because we were unable to obtain an informant from the vocational unit. To impute a reliable estimate of the expected number of ESs, we used a regression imputation technique. We included data on population size and the number of ESs from each vocational unit with complete data in linear regression models, treating the number of ESs as the dependent variable and the population size of the municipality where the IPS vocational units were located as the independent variable. For each of the four vocational units with missing ES data we calculated the expected number of ESs, using the constant and beta coefficients from the regressions, along with the population size of the vocational units with missing ES data (Supplementary File S2).

The data on the number of ESs was extrapolated to the population data for all Norwegian municipalities each year, enabling the calculation of the intensity of IPS for each municipality each year. For municipalities served by more than one IPS vocational unit, the intensity of each unit serving the municipality was summarized. These tables provide the underlying data for the annual heat maps showing IPS expansion across Norway. The heat maps were produced using Flourish, an online graphical software tool (Canva UK Operations Ltd, Hoxton Square London N1 6NN). Heat maps provide a means to illustrate incremental changes in the implementation of IPS over an 8-year period in a manner more easily interpreted than tables, not least since the number of municipalities in Norway during the study period was more than 400. Finally, we also aggregated data on geographical coverage of IPS, population coverage of IPS, intensity of IPS and population density to the county level to give an indication of how IPS was implemented at the regional level during the study period.

## Results

Table 1 provides an overview of the aggregated characteristics of the 73 IPS vocational units. In 2012, there were five operational IPS vocational units. On average, the number of ESs per vocational unit was 1.7 (SD 1.3). By 2019 the number of vocational units had increased to 55, and the number of ESs was on average 3.6 (SD 2.0).

**TABLE 1:** Characteristics of individual placement and support vocational units in 2012–2019 (Nordland, Norway, 2025).

Number of IPS vocational units per year	n	Mean (sd)
Number of IPS vocational units per year		
2012	5	
2013	11	
2014	12	
2015	14	
2016	19	
2017	21	
2018	37	
2019	55	
Average number of employment specialists per vocational unit in 2012		1.7 (1.3)
Average number of employment specialists per vocational unit in 2019		3.6 (2.0)

Geographical and population coverage of IPS, along with its intensity at the county level is provided in Supplementary File S3. In 2012, 4 out of 19 counties had IPS vocational units, covering between 3.9% and 69.7% of the municipalities within the counties. Population coverage was generally higher (14.8%), and the intensity of service provision was 1.0 ES/100,000. By 2019, IPS was present in all counties, with 49.4% of all municipalities having access to it. Population coverage was substantially higher, with 70.9% of the Norwegian population living in a municipality where IPS was provided. By 2019, the intensity of IPS had increased to 6.1 ES/100,000.

**FIGURE 1:**
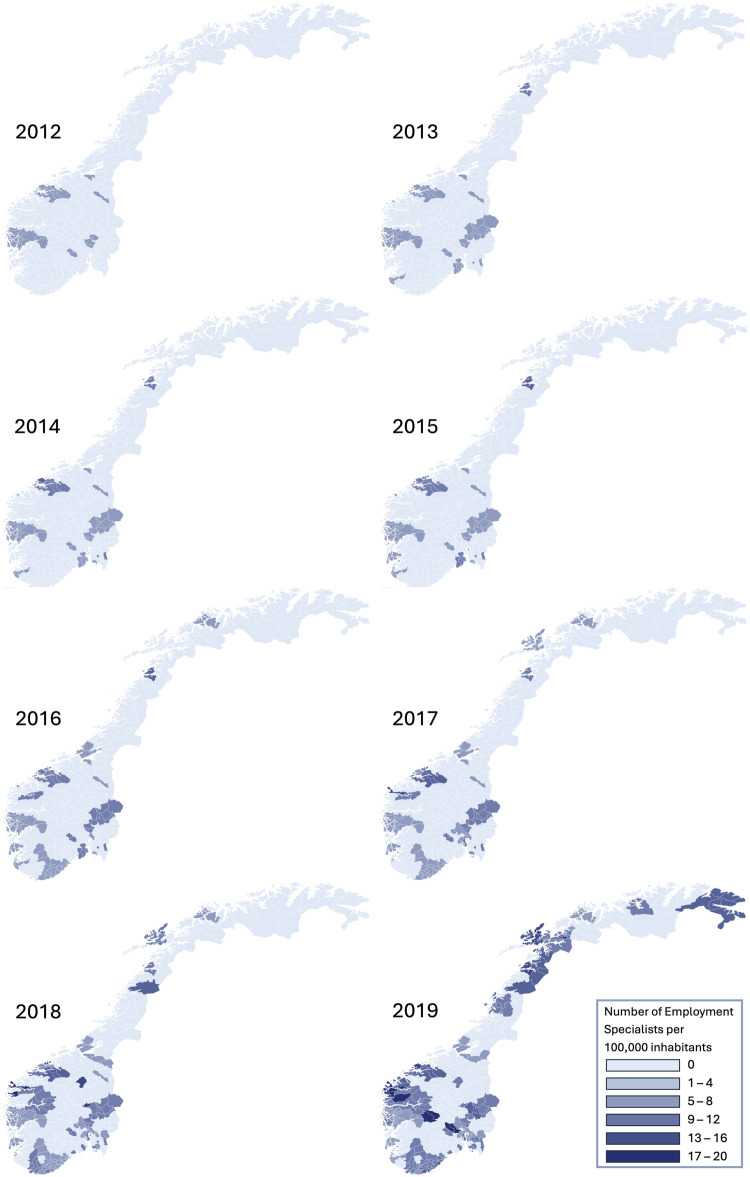
Intensity of individual placement and support in Norway from 2012 to 2019 (Nordland, Norway, 2025).

Figure 1 shows eight annual heat maps for IPS intensity per municipality. These maps illustrate the general pattern shown in Supplementary File S3 at a more detailed level. As can be seen in the early years, IPS was not available in the majority of municipalities. Over the years, IPS became available in new areas, but the intensity of service provision also increased substantially in areas where it was already established. By the end of our observation period, IPS had expanded to all major populated areas of Norway, and the intensity of service provision had increased to a substantially higher level than was the case initially.

## Discussion

Using a combination of qualitative and quantitative data we have presented the scaling up of IPS in Norway over a period of 8 years. To our knowledge, this is the first study to simultaneously present the nationwide growth of IPS over an 8-year period in terms of both geographic availability and intensity of provision. Our findings demonstrate that the process from proven efficacy, clinical initiatives and policy ambition to full scale implementation of a new service takes time and involves challenging processes. The efficacy of IPS has been demonstrated in several clinical trials and the service is now available on a large scale in Norway. The next logical step is to evaluate IPS in naturalistic effectiveness studies, which the present study has now paved the way for.

The link between geography and health is fundamental to epidemiology and has long been recognized as an important area for health service research and public planning. Perhaps the most famous example of how the concentration of events in a given geographical area can be used to understand a health problem is John Snow’s identification of the Broad Street pump as the epicenter of a cholera epidemic in 19th-century London [36]. More recently heat maps have been put to use for several purposes, with examples including studies of geographical differences in medication [37], access to invasive care in rural Canada [38] and mental health and education [39]. Although this is the first heat map of IPS growth to be published, there are some notable studies that are comparable and can provide context to our results. Mascayano and colleagues recently examined how IPS has expanded in the United States over the last decades and reported that the number of IPS programs now exceeds 1,000 [40]. Pogue et al. [41] conducted telephone interviews with mental health representatives from all 50 states in the United States, and examined the growth of IPS over a three-year period. They observed a substantial expansion of IPS services and found that IPS was available in 80% of states by 2019. Johnson-Kwochka et. al [42] calculated the state IPS program penetration rate as the number of IPS programs per 1 million inhabitants in the 50 US states. The authors observed rates ranging from 0.05 to 16.62 per 1 million individuals, which converts to 0.005-1.7 ES per 100,000 individuals. In England, national guidance states an aspiration of 1 IPS ES per 50,000 individuals in the general population, or 2 ES per 100,000 individuals in the general population [23]. These figures are calculations of service provision using the general population as the denominator. Applying a more specific target group as the denominator will of course have an impact on the ratio. As an example, Cram et. al calculated that the average intensity of ES was 3.7 per 10,000 individuals seen by a district health board’s specialist mental health and addiction services in New Zealand nationally. This would amount to 37 ESs per 100,000 individuals, but as the denominator is a more specific group than the general population the intensity of service provision is naturally higher [43]. Service intensity must also account for population density, as it reflects the geographical challenges of providing IPS in certain areas where ESs spend more time traveling to meet with clients. This is especially important in a country such as Norway, where distances, topography and weather conditions can obstruct access to support. The maps provided indicate that geographically, IPS is not present everywhere, but as is evident in Supplementary File S3, the service is available to the majority of the population, collectively illustrating how population density affects service provision. In terms of need versus capacity, the prevalence of individuals with mental illness at any given time varies according to the level of affluence and deprivation between regions [44]. As an example the prevalence of certain SMIs in Norway has been estimated to range between 1,200 and 2,300 per 100,000 individuals [45]. As eligibility for IPS is not only determined by age or diagnosis, it is challenging to extrapolate these figures to calculate the sufficient intensity of IPS service provision. Our results indicate that at a national level, the average IPS intensity was 6.1 ES *per capita* in 2019. Applying the England estimates for caseload per ES [23], this intensity corresponds to approximately 240–300 clients seen by an ES annually in a population of 100,000 individuals. Data from the Netherlands suggest that 2% of individuals using mental health services accessed IPS [46]. This rate is consistent with previous studies from the United States that found similar results within mental health services [47, 48]. However, as countries have expanded and scaled up IPS services, we now find access rates of around 9% in England [30] and Norway [29].

Several randomized controlled trials have been conducted on the effects of IPS, and the efficacy of the service is well documented [26]. It is however less certain whether a clinical service provided in a controlled setting will extend to a societal footprint when implemented in real-world settings. Consequently, understanding how the funding, scaling up and implementation of services unfold is imperative. Our results demonstrate substantial variation in the two key variables: the geographical availability of the service and the intensity of its provision. In any system where variance develops inequalities are naturally created, and in the case of IPS such inequalities undermine health equity. The practical consequence is that access to evidence-based care is highly dependent on area of residence, despite the service’s proven effectiveness and policy ambitions. It is difficult to pinpoint why such variation occurs, but prioritization within health services and funding schemes may be important factors. It is commonly recognized that implementation efforts are complex. How policies, stakeholders and processes interact can result in unpredictability, uncertainty and unintended consequences. A further challenge in scaling up services is balancing the goal of reaching the highest number of individuals with prioritizing those most in need. There are several obstacles to the successful implementation of IPS [24, 49, 50]. In the case of IPS implementation in Norway, funding for IPS became available in different formats and from different sources, leading to a case of organic stepwise growth rather than a planned and equal distribution across the country [16]. While IPS implementation was clearly anchored in policy ambition, it lacked a clear mandate and an official directive until recently. The development and growth of IPS in Norway has been detailed elsewhere, highlighting change agents, government use of research, funding, clarifications of sectoral interfaces and legal issues [16]. The empirical data collected in our study will enable us to study the consequences of this stepwise and organic form of growth, and the societal footprint of IPS in Norway.

### Limitations

This study has several strengths that contribute to the significance of our results and their implications. First, we conducted structured interviews with key IPS personnel to collect comprehensive data on the implementation of IPS throughout Norway. This ensured a nearly complete overview of every IPS vocational unit active during the study period, despite the absence of a central government funding scheme supporting their establishment. It should be noted that this form of data collection entails a risk of recall bias; however, the majority of the interviews took place within less than 5 years of the establishment of the vocational unit. We therefore believe that this risk is minimal. The number of ESs *per capita* is quite consistent across IPS vocational units, serving as a validation of the data extrapolated from the interviews. Population data was obtained from national population registries, which are reliable and complete. Finally, the visual display of results which combined both time and intensity allows readers to comprehend the gradual increase and growth of IPS across an entire country and how access to care expanded with increasing investments.

Our study also has some limitations. The intensity of IPS was quantified as the number of ESs *per capita* per geographical region, but the majority of the population will never be candidates for IPS support. In regions where a large proportion of inhabitants are not in the target group for IPS a low number of ESs may appear to indicate low service provision intensity, when in reality the service is adequately staffed. However, presenting healthcare resources on a *per capita* basis is common practice [51], and other alternatives such as limiting the denominator to individuals considered as the key target group for IPS are more difficult for resource planning as more detailed data are required. Finally, we would ideally have complemented the number of ESs with IPS fidelity as an indicator of the quality of IPS service provision. The majority of vocational units had indeed been subject to fidelity reviews, but we were unable to obtain them. However, according to a report on Norwegian IPS services from 2024, 95% of responding vocational units stated that they had been subject to an internal or external fidelity review [52].

### Conclusion

This study has quantified the geographic expansion and the increased intensity of IPS in Norway over an 8-year period, illustrating the journey from first initiatives to near population coverage. IPS expanded both geographically and in terms of intensity during the study period. Initially, 14.9% of the population lived in an area where IPS had been implemented, but by 2019 IPS was provided across the majority of Norway and 70.9% of the population lived in an area where IPS was available. Our study illustrates that the establishment and expansion of integrated employment support within mental health services is a gradual process: progressing from decision and ambition to full-scale implementation requires time and effort.

## Supplementary Material

SUPPLEMENTARY FILE S1 Interview guide individual placement and support method supervisor (Nordland, Norway, 2025).

SUPPLEMENTARY FILE S2 Imputation of individual placement and support intensity per year (Nordland, Norway, 2025).

SUPPLEMENTARY FILE S3 Individual placement and support service provision per county during 2012–2019 (Nordland, Norway, 2025).

## References

[1] Thomson JAK Tredennick H Barnes J. The Nicomachean Ethics. Penguin Publishing Group (2004). p. 400.

[2] Modini M Joyce S Mykletun A Christensen H Bryant RA Mitchell PB The Mental Health Benefits of Employment: Results of a Systematic Meta-Review. Australas Psychiatry (2016) 24(4):331–6. 10.1177/1039856215618523 26773063

[3] Boardman J Rinaldi M. Work, Unemployment and Mental Health. In: Ikkos G, editor. Social History of Psychiatry and Mental Health in Britain 1960–2010. Cambridge University Press (2021). p. 326–35.

[4] United Nations General Assembly. The Universal Declaration of Human Rights. New York: United Nations General Assembly (1948).

[5] United Nations. Convention on the Rights of Persons with Disabilities. New York: United Nations (2006).

[6] Latimer E Ray-Chaudhuri S Waters T. The Role of Changing Health in Rising Health-Related Benefit Claims. London: Institute for Fiscal Studies (2025).

[7] Ramsay CE Broussard B Goulding SM Cristofaro S Hall D Kaslow NJ Life and Treatment Goals of Individuals Hospitalized for First-Episode Nonaffective Psychosis. Psychiatry Res (2011) 189(3):344–8. 10.1016/j.psychres.2011.05.039 21708410 PMC3185187

[8] Iyer SN Mangala R Anitha J Thara R Malla AK. An Examination of Patient-Identified Goals for Treatment in a First-Episode Programme in Chennai, India. Early Interv Psychiatry (2011) 5(4):360–5. 10.1111/j.1751-7893.2011.00289.x 21951752 PMC3204158

[9] Adamus C Richter D Sutor K Zürcher SJ Mötteli S. Preference for Competitive Employment in People with Mental Disorders: A Systematic Review and Meta-Analysis of Proportions. J Occ Rehab (2024) 35:143–58. 10.1007/s10926-024-10192-0 38662329 PMC12089194

[10] Ernst W. Work, Psychiatry and Society, C. 1750-2015. Manchester, United Kingdom: Manchester University Press (2016). p. 398.

[11] Suijkerbuijk YB Schaafsma FG van Mechelen JC Ojajärvi A Corbière M Anema JR. Interventions for Obtaining and Maintaining Employment in Adults with Severe Mental Illness, a Network Meta-Analysis. Cochrane Database Syst Rev (2017) 9(9). 10.1002/14651858.CD011867.pub2 28898402 PMC6483771

[12] Kinoshita Y Furukawa TA Kinoshita K Honyashiki M Omori IM Marshall M Supported Employment for Adults with Severe Mental Illness. Cochrane Database Syst Rev (2013) 2013:CD008297. 10.1002/14651858.CD008297.pub2 24030739 PMC7433300

[13] Drake RE Becker DR Bond GR. Growth and Sustainment of Individual Placement and Support. Psychiatr Serv (2020) 71(10):1075–7. 10.1176/appi.ps.201900544 32746714

[14] Bond GR Peterson AE Becker DR Drake RE. Validation of the Revised Individual Placement and Support Fidelity Scale (IPS-25). Psychiatr Serv (2012) 63(8):758–63. 10.1176/appi.ps.201100476 22660842

[15] Sveinsdottir V Bull HC Evensen S Reme SE Knutzen T Lystad JU. A Short History of Individual Placement and Support in Norway. Psychiatr Rehabil J (2020) 43(1):9–17. 10.1037/prj0000366 30945917

[16] Moe C Brinchmann B Rasmussen L Brandseth OL McDaid D Killackey E Implementing Individual Placement and Support (IPS): The Experiences of Employment Specialists in the Early Implementation Phase of IPS in Northern Norway. The IPSNOR Study. BMC Psychiatry (2021) 21(1):632. 10.1186/s12888-021-03644-x 34930203 PMC8690340

[17] Norwegian Directorate of Health. Nasjonal Faglig Retningslinje for Utredning, Behandling Og Oppfølging Av Personer Med Psykoselidelser. In: Department of Psychiatric Care and Substance Abuse. Oslo, Norway (2013).

[18] Norwegian Ministry of Health and Care Services and the Norwegian Ministry of Labour and Social Inclusion. National Strategic Plan for Work and Mental Health 2007-2012 (2009). Available online at: https://www.regjeringen.no/globalassets/upload/hod/vedlegg/planer/i-1127eweb.pdf (Accessed June 24, 2024).

[19] Norwegian Ministry of Health and Care. Parliament Proposition Nr. [Om Opptrappingsplan for Psykisk Helse 1999 – 2006] (1997). Available online at: https://www.regjeringen.no/no/dokumenter/stprp-nr-63-1997-98-/id201915/ (Accessed June 24, 2024).63

[20] Norwegian Directorate of Health. National Guidelines: Individual Placement and Support (IPS) (2025). Available online at: https://www.helsedirektoratet.no/faglige-rad/individuell-jobbstotte-ips-og-helseiarbeid/individuell-jobbstotte-ips (Accessed August 13, 2025).

[21] Reme SE Monstad K Fyhn T Sveinsdottir V Løvvik C Lie SA A Randomized Controlled Multicenter Trial of Individual Placement and Support for Patients with Moderate-To-Severe Mental Illness. Scand J Work Environ Health (2019) 45(1):33–41. 10.5271/sjweh.3753 30074050

[22] Drake RE Bond GR Becker DR. Individual Placement and Support: An Evidence-Based Approach to Supported Employment. Oxford University Press (2012).

[23] NHS England. Individual Placement and Support for Severe Mental Illness. Guidance for Integrated Care Systems (2023). Available online at: https://www.england.nhs.uk/long-read/individual-placement-and-support-for-severe-mental-illness/ (Accessed March 24, 2025).

[24] Butenko D Rinaldi M Brinchmann B Brandseth OL Killackey E Mykletun A. The Personality Profile of IPS Employment Specialists, and How It Relates to Job Satisfaction: A Longitudinal Cohort Study. Scand J Psychol (2023) 64(1):71–9. 10.1111/sjop.12864 35997312 PMC10087514

[25] Butenko D Rinaldi M Moe C Brinchmann B Wittlund S Killackey E “What I Thought Was the Dream Job Was a Little Different Than I Had Expected”: A Qualitative Study Exploring the Turnover of IPS Employment Specialists. J Voc Rehab (2024) 61(1):79–91. 10.3233/jvr-240027

[26] Brinchmann B Widding-Havneraas T Modini M Rinaldi M Moe CF McDaid D A Meta-Regression of the Impact of Policy on the Efficacy of Individual Placement and Support. Acta Psychiatr Scand (2020) 141(3):206–20. 10.1111/acps.13129 31733146

[27] de Winter L Couwenbergh C van Weeghel J Sanches S Michon H Bond GR. Who Benefits from Individual Placement and Support? A Meta-Analysis. Epidemiol Psychiatr Sci (2022) 31:e50. 10.1017/S2045796022000300 35815640 PMC9281491

[28] Park AL Rinaldi M Brinchmann B Killackey E Aars NAP Mykletun A Economic Analyses of Supported Employment Programmes for People with Mental Health Conditions: A Systematic Review. Eur Psychiatry (2022) 65(1):e51. 10.1192/j.eurpsy.2022.2309 35983840 PMC9491084

[29] Brinchmann B Wittlund S Lorentzen T Moe C McDaid D Killackey E The Societal Impact of Individual Placement and Support Implementation on Employment Outcomes for Young Adults Receiving Temporary Health-Related Welfare Benefits: A Difference-In-Differences Study. Psychol Med (2024) 54(8):1787–95. 10.1017/S0033291723003744 38197145

[30] Rinaldi M Perkins R Baxter R Dorrington P Saville K. Individual Placement and Support (IPS): Duration of Employment Support and Equity of Access and Outcome in Routine Clinical Practice. B J Psych Bull (2024) 1–8. 10.1192/bjb.2024.68 39391936 PMC12676244

[31] Statistics Norway. Population Per Municipality in Norway in 2024. Available online at: https://www.ssb.no/statbank/table/07459/tableViewLayout1/ (Accessed July 7, 2024).

[32] World Bank Data. Washington DC: World Bank (2019). Available online at: https://data.worldbank.org/indicator/SI.POV.GINI?locations=NO (Accessed June 25, 2024).

[33] Brinchmann B. Can Individual Placement and Support (IPS) Contribute to Change the Life of Individuals Experiencing Mental Health Problems? Challenges Related to Efficacy, Effectiveness, and Implementation. [dissertation]. Tromsø (Norway): UiT-The Arctic University of Norway (2025).

[34] Malmberg-Heimonen I Tøge AG Bråthen M Frøyland K Spjelkavik Ø Bond G Assessing Program Fidelity Patterns within the IPS for Young Adults in Norway. J Evid Based Soc Work (2025) 1–16. 10.1080/26408066.2025.2528910 40611470

[35] Norwegian Government. Reform of Municipalities (2025). Available online at: https://www.regjeringen.no/no/dokument/dep/kdd/sak/saksgang-kommunereformen/id2607187/ (Accessed March 3, 2025).

[36] Newsom SW. Pioneers in Infection Control: John Snow, Henry Whitehead, the Broad Street Pump, and the Beginnings of Geographical Epidemiology. J Hosp Infect (2006) 64(3):210–6. 10.1016/j.jhin.2006.05.020 16891036

[37] Almonacid C Fitas E Sánchez-Covisa J Gutiérrez H Rebollo P. Geographical Differences in the Use of Oral Corticosteroids in Patients with Severe Asthma in Spain: Heat Map Based on Existing Databases Analyses. BMC Pulm Med (2023) 23(1):3. 10.1186/s12890-022-02295-2 36600236 PMC9812540

[38] Boyd JC Cox JL Hassan A Lutchmedial S Yip AM Légaré JF. Where You Live in Nova Scotia Can Significantly Impact Your Access to Lifesaving Cardiac Care: Access to Invasive Care Influences Survival. Can J Cardiol (2018) 34(2):202–8. 10.1016/j.cjca.2017.11.021 29407010

[39] Gao CX Clarke E Nicholas J Teo SM Koppe C Peter G Changes in Rates of Special Considerations in Higher Education Applications Pre- and during the COVID-19 Pandemic in Victoria, Australia. Early Interv Psych (2025) 19(1):e13603. 10.1111/eip.13603 39140403 PMC11730765

[40] Mascayano F Swanson S Florence AC Patel SR Amsalem D Pope LG Scaling up Evidence-Based Supported Employment in the United States. Psychiatr Serv (2025) 76(5):486–96. 10.1176/appi.ps.20240083 40167142

[41] Pogue JA Bond GR Drake RE Becker DR Logsdon SM. Growth of IPS Supported Employment Programs in the United States: An Update. Psychiatr Serv (2021) 73(5):533–8. 10.1176/appi.ps.202100199 34587785

[42] Johnson-Kwochka A Bond GR Becker DR Drake RE Greene MA. Prevalence and Quality of Individual Placement and Support (IPS) Supported Employment in the United States. Adm Policy Ment Health (2017) 44(3):311–9. 10.1007/s10488-016-0787-5 28062932

[43] Cram F Jury S Kokaua J Ku B Lockett H Wilson M. Individual Placement and Support (IPS) in Aotearoa New Zealand – New Insights from Linked Administrative Data. New Zealand: Ministry of Social Development (2020).

[44] Reilly S Olier I Planner C Doran T Reeves D Ashcroft DM Inequalities in Physical Comorbidity: A Longitudinal Comparative Cohort Study of People with Severe Mental Illness in the UK. BMJ Open (2015) 5(12):e009010. 10.1136/bmjopen-2015-009010 26671955 PMC4679912

[45] Norwegian Directorate of Health. Styringsinformasjon Til Helsefelleskapene. Del III: Pasienter Med Alvorlige Psykiske Lidelser. Oslo, Norway (2022).

[46] Vukadin M Zwinkels W Schaafsma F Spijkerman M de Graaf-Zijl M Delespaul P Effectiveness, Cost-Effectiveness and Return on Investment of Individual Placement and Support Compared with Traditional Vocational Rehabilitation for Individuals with Severe Mental Illness in the Netherlands: A Nationwide Implementation Study. BMJ Pub Health (2024) 2(1):e000393. 10.1136/bmjph-2023-000393 40018113 PMC11812774

[47] Bruns EJ Kerns SE Pullmann MD Hensley SW Lutterman T Hoagwood KE. Research, Data, and Evidence-Based Treatment Use in State Behavioral Health Systems, 2001-2012. Psychiatr Serv (2016) 67(5):496–503. 10.1176/appi.ps.201500014 26695495 PMC5107263

[48] Twamley EW Baker DG Norman SB Pittman JO Lohr JB Resnick SG. Veterans Health Administration Vocational Services for Operation Iraqi Freedom/Operation Enduring Freedom Veterans with Mental Health Conditions. J Rehabil Res Dev (2013) 50(5):663–70. 10.1682/jrrd.2012.08.0137 24013914

[49] Khalifa N Hadfield S Thomson L Talbot E Bird Y Schneider J Barriers and Facilitators to the Implementation of Individual Placement and Support (IPS) for Patients with Offending Histories in the Community: The United Kingdom Experience. Br J Occ Ther (2020) 83(3):179–90. 10.1177/0308022619879334

[50] Boardman J Rinaldi M. Difficulties in Implementing Supported Employment for People with Severe Mental Health Problems. Br J Psychiatry (2013) 203(3):247–9. 10.1192/bjp.bp.112.121962 24085736

[51] Saunes IS Karanikolos M Sagan A. Norway: Health System Review. Health Syst Transit (2020). 22, 1, 163. Available online at: https://eurohealthobservatory.who.int/publications/i/norway-health-system-review-20 (Accessed June 25, 2024). 32863241

[52] Samfunnsanalyse PROBA. Status for Individual Placement and Support (IPS) in Norway [Status for Individuell Jobbstøtte (IPS) I Norge] (2024). Oslo. Available online at: https://proba.no/rapporter/status-for-individuell-jobbstotte-ips-i-norge/ (Accessed: August 15, 2025).

