# Supplementary material for: Mapping the Growth of Individual Placement and Support Services in Norway
Source: Int J Public Health. 2025 Sep 29;70:1608739. doi: 10.3389/ijph.2025.1608739 (PMC12515731; doi:10.3389/ijph.2025.1608739)
Supplement: Supplementary file 1 [file DataSheet1.pdf]

Journal: International Journal of Public Health.

Title: Mapping the implementation of individual placement and support in Norway.

Supplementary file 1

Interview guide IPS Method Supervisor

Name

Phone / Email

How long has this person been a method supervisor?

Any previous method supervisor before this one? Name?

What is the catchment area for IPS in your area (area = the employment specialists you supervise)?

Current catchment area? (names of districts, municipalities, etc.)

When did you receive your first funding for IPS? Approximate date/year

When was the first employment specialist hired?

What was the reason for starting IPS in your area? (open question)

Have there been changes in the catchment area since the beginning? (Answer only since the start.)
What was the catchment area in 2012?
What was the catchment area in 2013?
What was the catchment area in 2014?
What was the catchment area in 2015?
What was the catchment area in 2016?
What was the catchment area in 2017?
What was the catchment area in 2018?
What was the catchment area in 2019?
What was the catchment area in 2020?
What was the catchment area in 2021?

How many full-time equivalent (FTE) employment specialists do you currently have?

How many FTE employment specialists have you had from 2012 until now? (Answer with approx. FTEs per year, e.g., 2.5)
2012?
2013?
2014?
2015?
2016?
2017?

2018?
2019?
2020?
2021?

Who is the target group?

- Common mental disorders (anxiety and depression)
- Moderate to severe mental disorders (bipolar disorder, schizophrenia, severe depression with psychosis)
- Substance use disorders
- Other (specify)

Any limitations on the target group? (For example, age group? Any prioritization?)

Do you accept referrals outside the target group?

Any adjustments to the target group over time?

Is IPS at your site part of a center for job mastery?

How is the IPS service organized today?
Where are the employment specialists employed? (number for each, or checkboxes)

- NAV
- Specialist health services
- Municipal health services
- Private company

Where are the employment specialists integrated? (number for each, or checkboxes)

- NAV
- Specialist health services
- Municipal health services
- Private company

Where do the employment specialists spend their time (when not visiting companies)? (none, some, a lot, most)

- NAV
- Specialist health services
- Municipal health services
- Private company

Who refers job seekers to IPS? (never, rarely, often)

- NAV

- Specialist health services
- Municipal health services
- Private company

Who is involved in the formal IPS collaboration in your catchment area
(multiple choices possible)

- NAV
- Specialist health services
- Municipal health services
- Private company

How are the positions financed today? (distribution in percentage for each)

- Project funding
- Regular operations

Do you have concerns related to the continued funding of IPS?

Are there problems with recruiting new employment specialists?

Is there anyone else we should talk to in order to answer these questions? Name, phone, email, workplace.

We are planning to conduct interviews/surveys with employment specialists. Would you be willing to be the contact person for further distribution of this?

Unit	Number of municipalities served by the vocational unit	Total population living in the municipalities covered by the vocational unit	Calculated IPS intensity
2015			
Vocational unit 1	3	>150,000	2.9
Vocational unit 2	5	>100,000	3.5
2016			
Vocational unit 1	3	>150,000	2.2
Vocational unit 2	5	>100,000	3.2
2017			
Vocational unit 1	3	>150,000	3.2
Vocational unit 2	5	>100,000	4.0
Vocational unit 3	1	>50,000	5.4
2018			
Vocational unit 1	3	>150,000	2.4
Vocational unit 2	5	>100,000	3.3
Vocational unit 3	1	>50,000	5.0
Vocational unit 4	1	<50,000	6.1
2019			
Vocational unit 2	5	>100,000	3.5
Vocational unit 3	1	>50,000	5.2
Vocational unit 4	1	<50,000	6.4

Supplementary file 2. Imputation of individual placement and support (IPS) intensity per year.

Supplementary file 3. Individual placement and support service provision per county during 2012 – 2019[a].

	2012			2013			2014			2015			2016			2017			2018			2019		
Counties	Geo cov	Pop cov	IPS	Geo cov	Pop cov	IPS	Geo cov	Pop cov	IPS	Geo cov	Pop cov	IPS	Geo cov	Pop cov	IPS	Geo cov	Pop cov	IPS	Geo cov	Pop cov	IPS	Geo cov	Pop cov	IPS
Finnmark	0.0 %	0.0 %	0	0.0 %	0,0 %	0.0	0.0 %	0,0 %	0,0	0.0 %	0.0 %	0.0	0.0 %	0.0 %	0.0	0.0 %	0.0 %	0.0	0.0 %	0.0 %	0.0	42.0 %	60.5 %	9.6
Troms	0.0 %	0.0 %	0	0.0 %	0.0 %	0.0	0.0 %	0,0 %	0,0	0.0 %	0.0 %	0.0	4.2 %	44.7 %	1.9	4.2 %	45.0 %	1.6	4.2 %	45.4 %	2.4	33.0 %	67.6 %	5.5
Nordland	0.0 %	0.0 %	0	2.3 %	20.5 %	4.1	2.3 %	20.6 %	7.2	2.3 %	20.8 %	8.6	2.3 %	20.9 %	8.3	15.9 %	34.4 %	3.5	18.2 %	45.4 %	7.1	54.5 %	77.0 %	9.9
Nord-Trøndelag	0.0 %	0.0 %	0	0.0 %	0.0 %	0.0	0.0 %	0.0 %	0.0	0.0 %	0.0 %	0.0	4.3 %	5.0 %	1.2	4.3 %	5.0 %	1.2	-		-	-		-
Sør-Trøndelag	8.0 %	63.5 %	2.4	4.0 %	59.4 %	0.6	4.0 %	59.5 %	3.3	4.0 %	59.7 %	0.5	24.0 %	66.3 %	1.7	24.0 %	66.4 %	1.2	-		-	-		-
Trøndelag	-		-	-		-	-		-	-		-	-		-	-		-	21.3 %	53.0 %	2.7	18.8 %	52.0 %	3.9
Oppland	0.0 %	0.0 %	0	19.2 %	30.2 %	1.8	19.2 %	30.3 %	3.5	19.2 %	30.3 %	3.5	19.2 %	30.4 %	5.2	19.2 %	30.4 %	6.1	23.1 %	45.2 %	7.3	23.1 %	45.3 %	6.7
Hedmark	0.0 %	0.0 %	0	36.4 %	64.5 %	2.8	36.4 %	64.6 %	2.8	36.4 %	64.9 %	3.6	36.4 %	65.1 %	4.3	36.4 %	65.4 %	4.3	40.9 %	66.4 %	5.3	40.9 %	66.7 %	7.4
Akershus	0.0 %	0.0 %	0	9.1 %	9.8 %	4.1	9.1 %	9.7 %	4.0	9.1 %	9.6 %	4.0	9.1 %	10.5 %	3.9	9.1 %	30.6 %	4.5	40.9 %	57.7 %	2.0	63.6 %	75.9 %	2.2
Østfold	0.0 %	0.0 %	0	38.9 %	57.3 %	3.1	44.4 %	44.9 %	3.8	44.4 %	58.3 %	4.8	44.4 %	56.2 %	5.5	50.0 %	46.4 %	6.0	61.1 %	65.4 %	5.1	61.1 %	65.5 %	5.7
Oslo	0.0 %	0.0 %	0	0.0 %	0.0 %	0.0	17.6 %	20.9 %	2.3	17.6 %	20.9 %	3.0	100.0 %	100.0 %	0.6	100.0 %	100.0 %	0.9	100.0 %	100.0 %	1.9	100.0 %	100.0 %	3.6
Vestfold	0.0 %	0.0 %	0	50.0 %	77.0 %	1.6	50.0 %	76.9 %	3.2	50.0 %	76.9 %	4.3	50.0 %	77.0 %	4.2	8.3 %	2.7 %	2.3	22.2 %	27.8 %	3.9	22.2 %	27.9 %	4.2
Buskerud	0.0 %	0.0 %	0	0.0 %	0.0 %	0.0	0.0 %	0.0 %	0.0	0.0 %	0.0 %	0.0	0.0 %	0.0 %	0.0	19.0 %	53.2 %	2.3	19.0 %	53.2 %	2.2	33.3 %	60.2 %	9.3
Telemark	0.0 %	0.0 %	0	0.0 %	0.0 %	0.0	0.0 %	0.0 %	0.0	0.0 %	0.0 %	0.0	0.0 %	0.0 %	0.0	0.0 %	0.0 %	0.0	5.6 %	8.2 %	7.1	5.6 %	8.1 %	7.1
Aust-Agder	0.0 %	0.0 %	0	0.0 %	0.0 %	0.0	0.0 %	0.0 %	0.0	0.0 %	0.0 %	0.0	100.0 %	100.0 %	0.2	100.0 %	100.0 %	1.7	100.0 %	100.0 %	2.6	100.0 %	100.0 %	7.2
Vest-Agder	0.0 %	0.0 %	0	0.0 %	0.0 %	0.0	0.0 %	0.0 %	0.0	0.0 %	0.0 %	0.0	46.7 %	78.4 %	2.8	46.7 %	78.5 %	3.1	93.3 %	99.5 %	4.5	93.3 %	99.5 %	5.5
Rogaland	0.0 %	0.0 %	0	15.4 %	14.2 %	1.6	15.4 %	14.3 %	1.5	42.3 %	71.6 %	2.6	42.3 %	57.1 %	2.3	26.9 %	57.0 %	3.6	26.9 %	56.9 %	2.9	42.3 %	69.6 %	2.6
Hordaland	69.7 %	85.9 %	0.4	69.7 %	86.0 %	0.4	69.7 %	86.1 %	0.8	72.7 %	87.3 %	0.9	72.7 %	87.4 %	2.0	72.7 %	87.4 %	2.2	72.7 %	87.5 %	2.7	72.7 %	87.6 %	3.2
Sogn og Fjordane	3.9 %	2.1 %	0.4	3.8 %	2.1 %	0.4	3.8 %	2.1 %	0.8	3.8 %	2.1 %	0.8	26.9 %	38.4 %	4.6	26.9 %	38.3 %	4.6	57.7 %	64.5 %	5.7	92.3 %	98.2 %	6.2
Møre og Romsdal	27.8 %	27.4 %	1.4	27.8 %	27.4 %	1.4	27.8 %	27.3 %	7.0	33.3 %	38.8 %	4.9	33.3 %	38.7 %	5.8	33.3 %	38.6 %	8.3	36.1 %	56.3 %	8.1	45.7 %	59.1 %	10.6
Total	9.6 %	14.8 %	1.0	16.2 %	24.6 %	1.5	16.4 %	26.0 %	2.9	18.7 %	31.2 %	3.0	29.9 %	52.7 %	2.8	30.1 %	53.9 %	3.3	37.5 %	63.2 %	4.1	49.4 %	70.9 %	6.1

[a]: Geo. cov: geographical coverage of individual placement and support (IPS), percentage of municipalities within the county covered by IPS vocational unit (s). Pop. cov: population coverage of IPS, percentage of population within the county living in a municipality where IPS is available. IPS: Average intensity of IPS (employment specialists/100.000 inhabitants) in the municipalities within the county where IPS is available.
